# Grinspan Syndrome Associated With Pemphigus Vulgaris Induced by COVID-19 Vaccination

**DOI:** 10.7759/cureus.88255

**Published:** 2025-07-18

**Authors:** Anass Saddik, Fouzia Hali, Soumiya Chiheb, kaoutar Elkhalifa, Ihsane Benyahya

**Affiliations:** 1 Department of Dermatology and Venereology, Hassan II University, Casablanca, MAR; 2 Department of Surgical Odontology, Hassan II University, Casablnaca, MAR; 3 Department of Surgical Odontology, Hassan II University, Casablanca, MAR

**Keywords:** covid 19, grinspan syndrome, oral lichen planus, pemphigus vulgaris, vaccination

## Abstract

Grinspan syndrome (GS) is characterized by the association of diabetes mellitus, hypertension, and lichen planus. It remains enigmatic to determine whether this represents a distinct pathology or if it is a lichenoid reaction secondary to the use of medications for treating hypertension and diabetes. Oral lichen planus (OLP) is a common mucocutaneous disease that can affect the skin, mucous membranes, or both. An association with pemphigus vulgaris remains exceptional. We describe a rare case of GS associated with pemphigus vulgaris induced by COVID-19 vaccination. An 80-year-old female with type 2 diabetes mellitus and vascular hypertension was admitted to our department for bullous dermatosis.

## Introduction

Grinspan syndrome (GS) is an extremely rare triad condition that includes oral lichen planus (OLP, with or without skin involvement), diabetes mellitus, and arterial hypertension [1].

Pemphigus is a rare autoimmune blistering disease, reported to be associated with other coexisting and autoimmune diseases. The combination of pemphigus and lichen planus (LP) is very rare [2].

LP is defined as a chronic inflammatory dermatosis that can affect the skin and mucous membranes or both [3].

COVID-19 caused by SARS-CoV-2 was reported in December 2019 and then became a pandemic in March 2020. Faced with the serious problems linked to this COVID-19 pandemic, vaccines against SARS-CoV-2 emerged as a solution. However, several adverse effects have been reported, including mucocutaneous manifestations [3].

We report here a rare case of oral LP associated with pemphigus vulgaris (PV) shortly after COVID-19 vaccination in an 80-year-old diabetic and hypertensive woman.

## Case presentation

**Figure 1:**
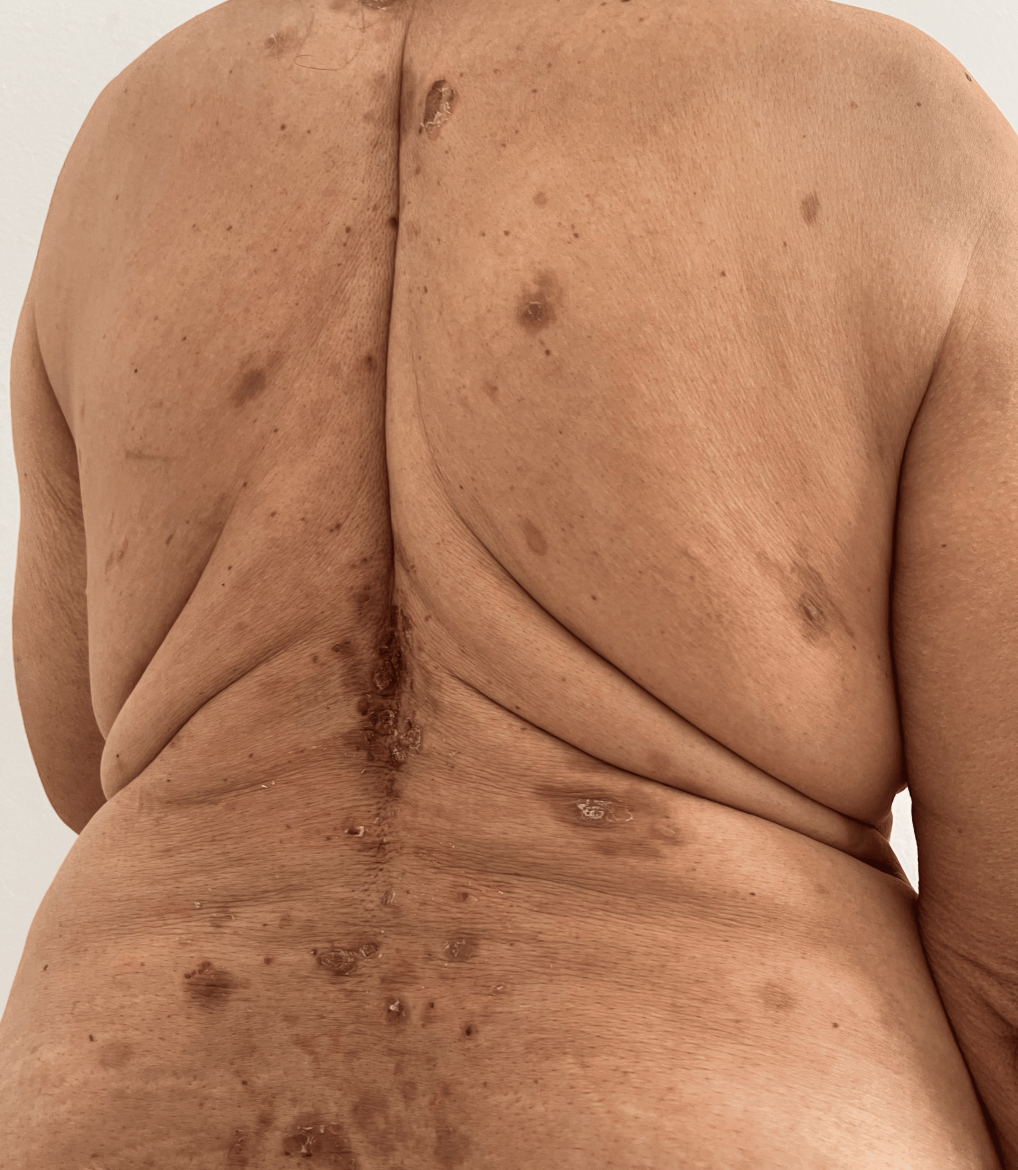
Post-bullous erosions of different sizes.

An 80-year-old patient, diabetic and on oral antidiabetics, hypertensive with monotherapy, and cholecystectomized 15 years ago, presented one month after receiving the second dose of the Oxford-AstraZeneca COVID-19 vaccine with extensive bullous lesions. Preceded by pruritus, these lesions progressed to post-bullous erosions, affecting the oral and genital mucous membranes. The clinical examination found post-bullous erosions of different sizes, impetiginized in places with positive Nikolsky (Figure 1).

**Figure 2:**
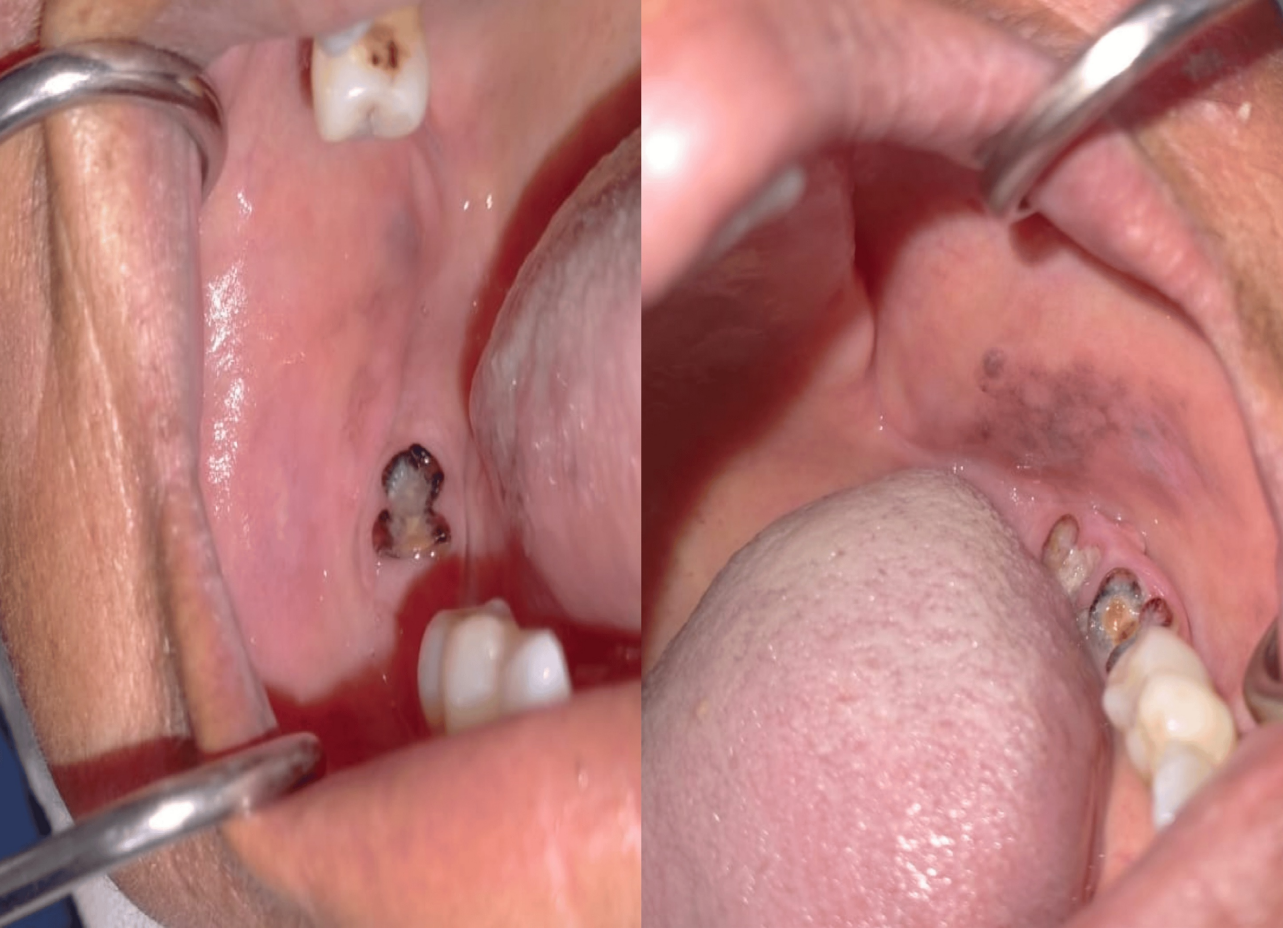
Two poorly limited bilateral pigmented lesions.

In addition, the examination of the oral mucosa showed two poorly limited bilateral pigmented lesions with a poor oral condition (Figure 2).

**Figure 3:**
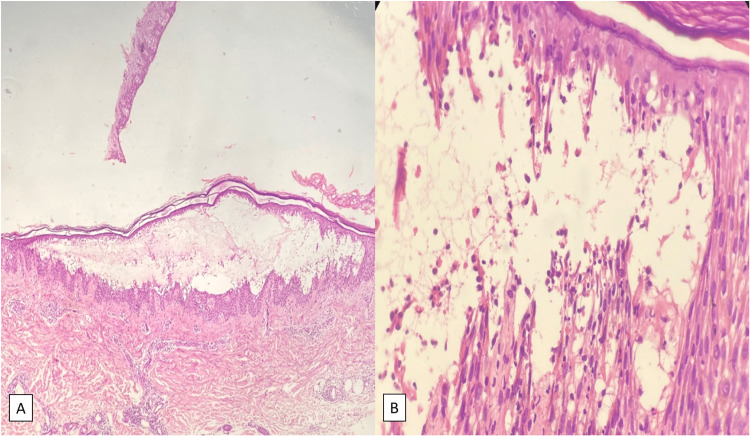
On microscopic examination, an intraepidermal bubble with acantholysis is observed, containing keratinocytes, neutrophils, and eosinophils: (A) magnification x4; (B) magnification x40.

The diagnosis of pemphigus was confirmed through a skin biopsy (Figure 3) and direct and indirect immunofluorescence testing, revealing positive anti-intercellular space autoantibodies at 40, compared to a normal value of less than 20.

After dental consultation and oral medicine evaluation at the Surgical Odontology Department within the Dental Consultation and Treatment Center of Casablanca University Hospital, the diagnosis of OLP was confirmed. This was further supported by a cheek biopsy, revealing its lichen nigricans form. Viral serologies (HIV and hepatitis) were negative.

**Figure 4:**
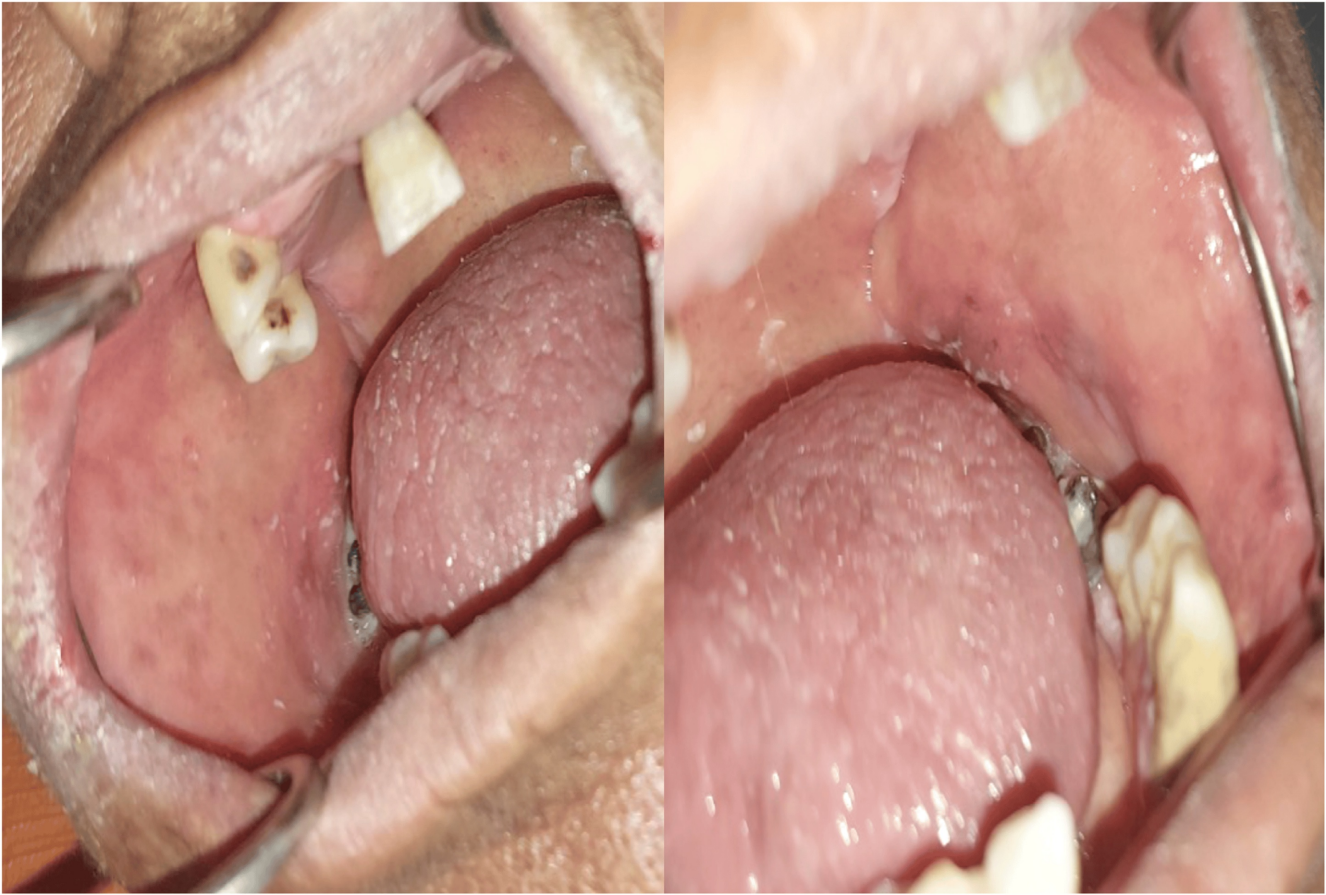
Clear regression of oral mucosal lesions.

We confirmed the diagnosis of GS (OLP, diabetes mellitus, and arterial hypertension) associated with PV triggered by the COVID-19 vaccine. Therapeutically, the patient was put on oral corticosteroid therapy 1 mg/kg/day (70 mg/day) with azathioprine-type immunosuppressant. Currently, we have initiated a reduction at the rate of 5 mg every three weeks, observing skin whitening and a slight regression of oral lichen (Figure 4). In addition, the patient was put on rapid insulin according to capillary blood glucose after starting corticosteroid therapy.

## Discussion

OLP can appear clinically in various forms (reticular, erosive and ulcerated, hypertrophic, atrophic, bullous, or pigmented). LP nigricans or the pigmentary form is a rare variant of LP of unknown etiology, probably due to the overproduction of melanin [4].

This pathology generally affects women more than men with an average age at diagnosis of 55 years [3].

It is also noted that oral lichen most times is asymptomatic; otherwise, it can manifest itself by dryness, burns, or irritation [5]. The involvement of the mucous membranes is often multifocal and symmetrical affecting the endojugal mucosa and the tongue [4].

GS is a rare clinical entity, characterized by the triad of OLP, diabetes mellitus, and hypertension. Some theories incriminate drugs used to treat diabetes and hypertension in these lichenoid reactions of the oral mucosa [1].

Pemphigus is an autoimmune disease caused by circulating autoantibodies against keratinocyte cells in the skin or mucous membranes. Although many combined cases of pemphigus and other autoimmune diseases have been reported, the coexistence of pemphigus and LP remains rare [2].

Neumann-Jensen et al. described the first three cases of the association of LP and pemphigus, confirmed clinically, histopathologically, and immunologically [6].

We also found an unusual case of association of periorbital pigmentary LP and PV, treated with a combination of oral corticosteroid therapy and azathioprine, as in the case of our patient [7].

In the literature, there is only one case of the coexistence of PV and LP following vaccination against COVID-19 in a 43-year-old man. According to several studies, COVID-19 vaccines could trigger autoimmune diseases such as PV [8].

In our case, the simultaneous occurrence of the two pathologies, OLP and pemphigus, was significant and may suggest a potential causal link between the two conditions in terms of pathophysiology. The coexistence of these two diseases could also be a coincidence since it is rare. Further studies need to be performed to investigate the possible relationship between OLP and PV.

## Conclusions

Nigricans OLP is a rare variant of LP. The coexistence of OLP and PV suggests that there could be a relationship between these two pathologies in terms of immunological mechanisms. Although the development of GS after vaccination against COVID-19 may be coincidental, further studies on a large sample are needed.
